# Effects of technology-assisted rehabilitation for patients with hip arthroplasty: A meta-analysis

**DOI:** 10.1097/MD.0000000000035921

**Published:** 2023-11-10

**Authors:** Lingyun Wu, Xiaoyan Li, Lijiangshan Hua, Qiuhua Sun

**Affiliations:** a School of Nursing, Zhejiang Chinese Medical University, Hangzhou, China.

**Keywords:** hip arthroplasty, meta-analysis, rehabilitation, telerehabilitation, virtual reality

## Abstract

**Background:**

To investigate the efficacy of technology-assisted rehabilitation compared to that of usual care programs after total hip arthroplasty (THA) through randomized controlled trials (RCTs).

**Methods:**

The Medline (PubMed), Cochrane Library, Embase and Web of Science databases were searched for RCTs regarding the efficacy of technology-assisted rehabilitation following THA. Data were analyzed using Stata 12.0 software.

**Results:**

Eleven RCTs involving 1327 patients were included in the meta-analysis. The pooled effect size showed that compared to usual care, telerehabilitation significantly improved the Harris score (standardized mean difference [SMD] 0.74, 95% confidence interval [CI] 0.58 to 0.90) and functional independence measure (FIM) score (SMD 1.26, 95% CI 0.48 to 2.03). In addition, video-based therapy could significantly improve walk test results (SMD 0.43, 95% CI 0.11 to 0.75).

**Conclusion:**

The findings suggest that technology-assisted rehabilitation, especially telerehabilitation, have been shown to improve the physical function of patients following THA compared to conventional rehabilitation. More robust studies are needed to validate the long-term efficacy and safety of innovative technology-assisted training strategies.

## 1. Introduction

Total hip arthroplasty (THA) is a common reconstructive hip surgery that is performed on patients who do not respond to long-term medication and conservative treatment to alleviate their pain and joint stiffness.^[1,2]^ As the population ages, the average age of patients treated decreases, and with higher expectations for quality of life, the number of hip replacement operations continues to increase each year.^[3]^ Rehabilitation is a multidisciplinary approach that improves joint function and activities of daily living, as well as reducing pain for the individual after surgery.^[4]^ Several studies have demonstrated the effectiveness of rehabilitation after hip replacement surgery.^[5–7]^ Rehabilitation therapy, for example, can promote functional recovery and reduce adverse complications such as dislocations of prosthetics and deep vein thrombosis.^[8,9]^

Technology and science have been advancing rapidly in recent years, and which led to frequent changes in the methods and measures of rehabilitation. Therefore, new technology-assisted training strategies can be applied to guide the rehabilitation of patients after arthroplasty, aimed at improving their motor function and activities of daily living. One definition of eHealth is “the use of digital information and communication to support or improve health and health care services.”^[10]^ eHealth interventions include internet-based interventions, telephone support, virtual reality, exercise games, and mobile applications. In recent years, eHealth has been utilized to facilitate the management of chronic diseases, cardiac rehabilitation, musculoskeletal disorders, cancer, and neonatal care improve the health outcomes of patients.^[11–14]^ eHealth can also improve access to treatment, reduce waiting times, and be more cost-effective than face-to-face interventions.^[15]^

Despite systematic reviews of technology-assisted rehabilitation, there is insufficient evidence to support the effectiveness of this method in the rehabilitation of THA patients. Additionally, there is no consensus regarding the impact of technology-assisted rehabilitation on the management of hip replacement due to conflicting findings and high heterogeneity between studies. Therefore, the objective of this meta-analysis was to determine the effectiveness of technology-assisted rehabilitation in THA rehabilitation versus conventional rehabilitation care.

## 2. Materials and methods

This study was performed according to the standard methodology outlined in the Cochrane Handbook and PRISMA (Preferred Reporting Items for Systematic Reviews and Meta-analyses) checklist. Ethical approval was unnecessary in this study because the analyses were based on previously published studies.

### 2.1. Data sources

The Medline (PubMed), Cochrane Library, Embase and Web of Science databases were searched by 2 reviewers. The MeSH terms and keywords were used to generate a strategy for the following terms: telecare, remote rehabilitation, virtual rehabilitation, telerehabilitation, virtual reality, exergames, games, video, eHealth, mobile health, internet-based intervention, hip replacement, hip arthroplasty, random, random allocation, and randomized controlled trials. The databases were searched from their inception to March 1, 2023. The detailed retrieval strategies were provided in Supplemental Table 1, http://links.lww.com/MD/K623. In addition, the reference lists of the included studies, present meta-analyses, and systematic reviews were searched manually for any missed studies.

### 2.2. Eligibility criteria

Eligible studies were considered if they met the following criteria: PICOS (population, intervention, comparator, outcome, study design). Eligible studies were identified by following the PICOS (Participants, Interventions, Comparisons, Outcomes and Study design) principle. Participants: patients undergoing rehabilitation after THA. Intervention: the experiment group received any eHealth interventions. Comparison: the control group received usual care or no treatment. Outcomes: available data about Harris score, functional independence measure (FIM), timed up-and-go test (TUG), Hip Dysfunction and Osteoarthritis Outcome Score for Joint Replacement (HOOS JR), walk test, activities of daily living (ADL), mental, and physical health were reported. The Harris score, TUG, HOOS JR, and walk test were used to assess physical function. The FIM, ADL, mental health, and physical health were used to assess independence level and quality of life (QOL). Study design: Randomized controlled trials. The exclusion criteria were as follows: unavailable data, case reports, animal studies, reviews, and duplicated publications. Any disagreements between the 2 reviewers were resolved by a third reviewer.

### 2.3. Data extraction and quality assessment

Two authors independently collected available data from the eligible articles using a standard data extraction form. The extracted data included the first author name, publication year, age, gender, sample size, intervention method, outcomes, and follow-up. Any disagreements between the 2 reviewers were resolved by a third reviewer. The methodological quality of the randomized controlled trials (RCTs) was accessed using the Cochrane risk-of-bias tool. The tool contains 6 items, including random sequence generation, allocation concealment, blinding of participants and personnel, blinding of outcome assessment, incomplete outcome data and selective reporting, and other biases.

### 2.4. Statistical analysis

We conducted the meta-analysis using STATA 12.0 (Stata Corp, College Station, Texas). The *I*^2^ test and Cochrane χ^2^ statistic were used to quantify heterogeneity among the studies. A chi-squared *P* ≤ .05 and an *I*^2^ > 50% indicated substantial heterogeneity among the studies and a random-effects model was used. A chi-squared *P* > .05 and an *I*^2^ ≤ 50% indicated that there was no obvious heterogeneity among the studies, and a fixed-effects model was used. For continuous data, the standardized mean difference (SMD) and 95% confidence interval (CI) were calculated. Potential publication bias was assessed by Begg and Egger tests. Subgroup analysis was used to explore the origins of heterogeneity if applicable.

## 3. Result

### 3.1. Literature search and characteristics of the included studies

**Figure 1.**
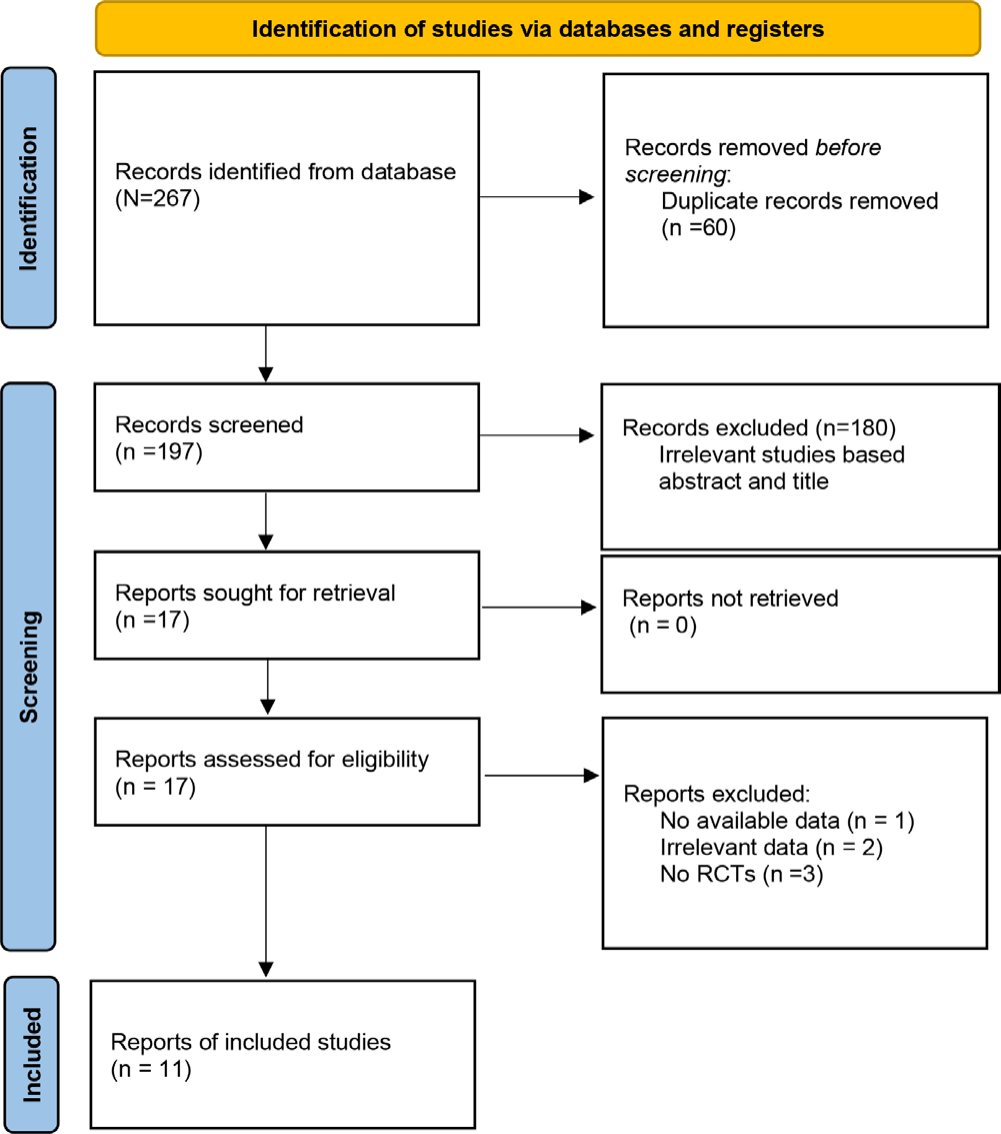
Flowchart of the study procedure.

The PRISMA 2020 flowchart was used to represent the selection process (Fig. 1). A total of 309 articles were retrieved from the databases via the search strategy. Seventy-eight were removed due to duplication. A total of 231 articles were retained after screening the title and abstract. After the full text was assessed, 10 articles were excluded because they did not conform to the eligibility criteria. Finally, 11 studies^[16–26]^ were included in this study. Among these studies, a total of 1327 patients were included; there were 634 cases in the intervention group and 693 patients in the control group. The median age of the included patients ranged from 53.3 to 77 years. The sample size varied from 15 to 200. Technology-assisted interventions have been applied in different studies. Eight studies^[16,19–25]^ had intervention groups that received telerehabilitation. Three studies^[17,18,26]^ investigated video-assisted rehabilitation. The characteristics of the included studies are described in Table 1.

**Table 1:** Characteristics of the studies.

Studies	Country	Age	Sample size	Gender (F/M)	Intervention	Follow-up
Li 2014	China	T: 66.2 ± 15.5 C: 67.3 ± 16.7	T: 100 C: 137	T: 54/46 C: 84/53	T: patients were contacted by telephone 3 to 7d after discharge and 1 and 3 mo after discharge. Depending on joint and muscle function at different stages, patients received individualized health education and guidance including exercise, cautions in daily life, and regular examination accordingly. Each call lasted 20–30 min. C: patients received conventional discharge guidance and follow-up.	6m
Hørdam 2009	Denmark	T: 75 C: 74.8	T: 68 C: 93	T: 47/21 C: 58/35	T: patients received telephone support and counseling 2 and 10 wk after surgery. C: patients received the standard postoperative procedure in the hospital, which means discharge after 5 to 7 d and a clinical control in the outpatient department after 3 mo	9m
Nelson 2020	Australia	T: 62 ± 9 C: 67 ± 11	T: 35 C: 35	T: 23/12 C: 21/14	T: patients was identical in content to the control programme, with the mode of delivery being via telerehabilitation technology on an Apple iPadTM into participants’ homes C: patients received usual care.	6w
Anthony 2022	USA	T: 65 C: 63	T: 45 C: 45	T: 11/34 C: 13/31	T: patients received automated mobile phone text messages twice a day (morning and evening) communicating an ACT-based intervention for 14 d with d 1 starting the d after enrollment into the study. C: patients received the same morning and evening messages during the 2-wk study period.	2w
Zhang 2022	China	T: 77 ± 7.89 C: 75.17 ± 7.73	T: 27 C: 24	T: 17/10 C: 16/8	T: patients received a “home-oriented post-operative rehabilitation management system for geriatric hip fractures” developed by our department, was used for home-based telerehabilitation. C: the patients received telephone follow-ups at 2 weeks, 1 mo, 2 mo, and 3 mo after discharge from the hospital.	3m
Wang 2018	China	T: 77 ± 7.89 C: 75.17 ± 7.73	T: 200 C: 200	T: 17/10 C: 16/8	T: Besides routine continuous nursing methods (telephone follow-up within 1 mo after discharge, outpatient review at 1, 3 and 6 mo after discharge), the patients in the intervention group interacted with the same nurse specialist in the platform C: patients received usual care.	6m
Eichler 2019	Germany	T: 53.3 ± 7 C: 56.8 ± 5.7	T: 48 C: 39	T: 26/22 C: 19/20	T: patients assigned to the IG performed a 3-mo, home-based telerehabilitation program based on the MeineReha system, which consisted of a home component as well as a working portal for the therapist in the clinic. C: patients received usual care.	3m
Kalron 2018	Israel	T: 65.7 ± 7.8 C: 67.3 ± 9.5	T: 15 C: 17	N/A	T: patients received a video platform for therapy software program. C: patients received an exercise booklet with exercises similar to those of the telerehabilitation group.	6w
Tappen 2003	USA	T: 75.28 ± 9.48 C: 69.61 ± 12.65	T: 39 C: 43	NA	T: The intervention consisted of 2 parts: video-taping the study subjects during their physical therapy sessions and the showing 1 of 2 generic educational videos that were produced for this study. C: patients received usual care.	3m
Zavala-González 2022	Chile	T: 69.6 ± 8.8 C: 69.9 ± 8.8	T: 36 C: 37	T: 18/18 C: 20/17	T: patients received the same program as the control group, plus 10 min of virtual reality through the Wii Fit plusTM application. C: patients received usual physiotherapy treatment.	6w
Fascio 2022	Italy	T: 61.5 ± 6.21 C: 60.9 ± 7.52	T: 21 C: 23	T: 9/12 C: 10/13	T: patients received the same program as the control group, plus 30 min of 2.4. Virtual Reality Rehabilitation System. C: patients received usual home exercise program.	15d

C = control, F = female, M = male, T = treatment.

### 3.2. Quality assessment of the included studies

**Figure 2.**
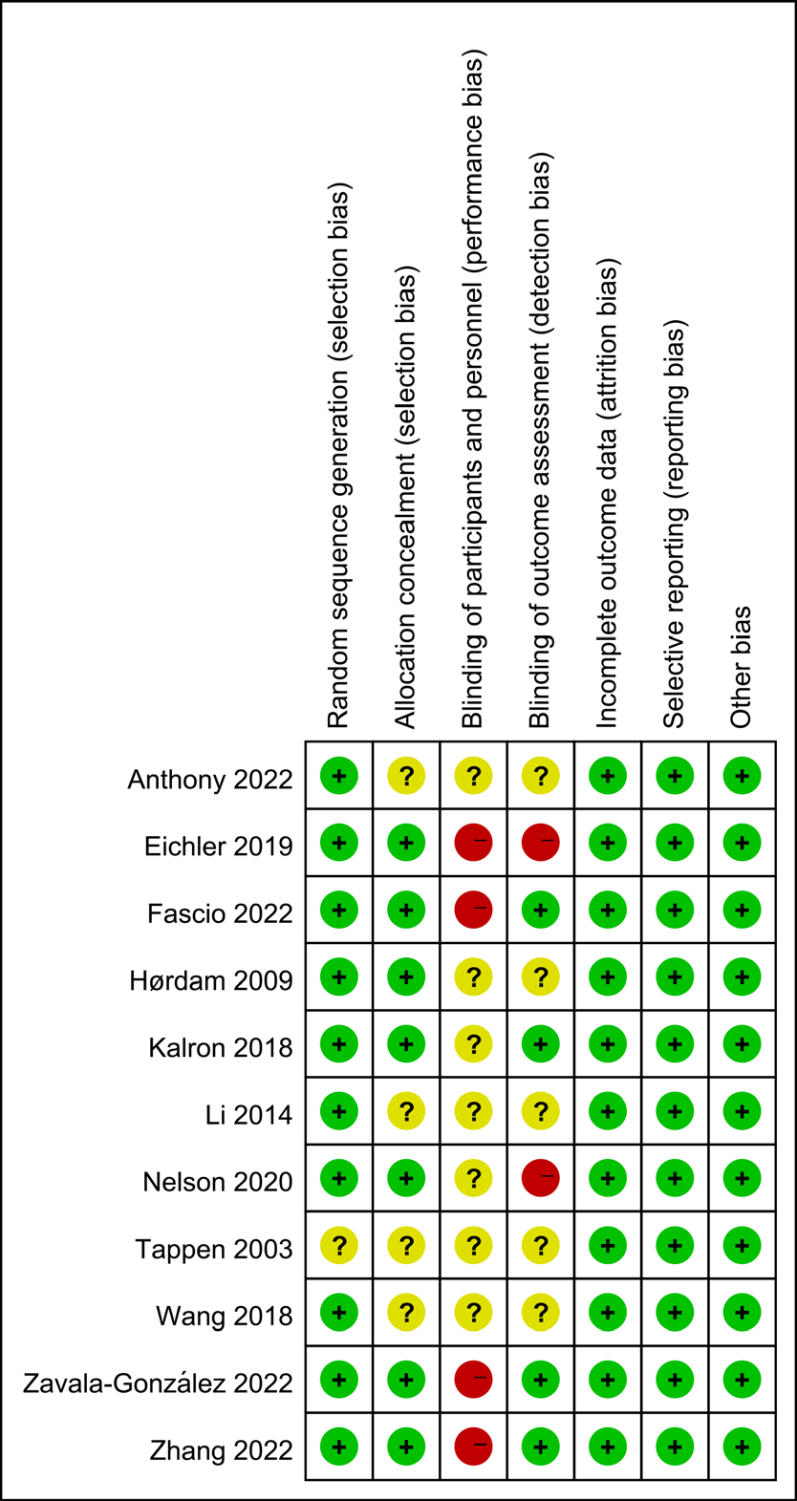
Risk of bias of the included studies.

Based on the Cochrane Collaboration recommendation, the quality assessment of the included studies is summarized in Figure 2. Ten RCTs^[16–25]^ reported random sequence generation. Seven RCTs^[16–18,20,21,23,25]^ reported allocation concealments. Four RCT^[16–18,23]^ outcome assessors were blinded. In addition, details of the blinding of participants and personnel were not described in all studies and 4 studies^[16–18,21]^ had a high risk of bias. All studies had a low risk of incomplete outcome data and selective reporting, and other biases.

### 3.3. Outcomes

#### 3.3.1. Harris score.

**Figure 3.**
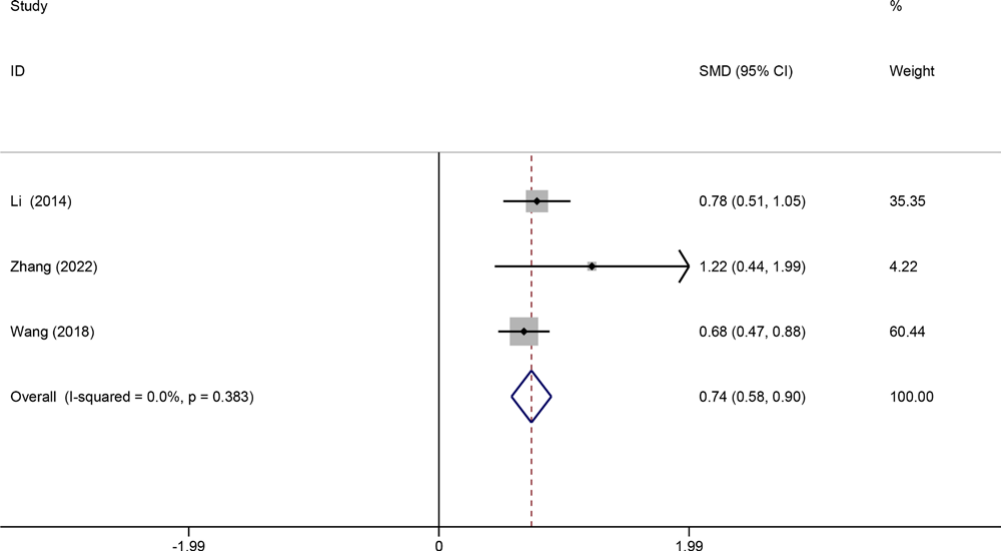
Forest plot of Harris. CI = confidence interval, SMD = standardized mean difference.

Three studies measured the effects of telerehabilitation on the Harris score. The pooled meta-analysis showed that telerehabilitation significantly improved the Harris score (SMD 0.74, 95% CI 0.58 to 0.90, *P* = .001, *I*^2^ = 0%) (Fig. 3).

#### 3.3.2. FIM.

**Figure 4.**
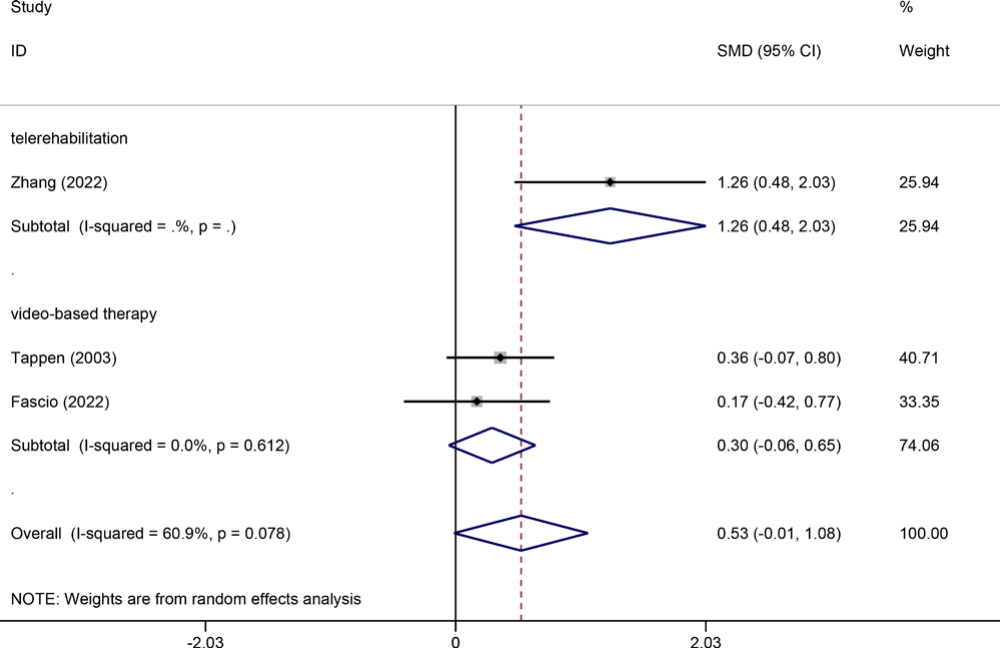
Forest plot of FIM. CI = confidence interval, FIM = functional independence measure, SMD = standardized mean difference.

Three studies were included in the meta-analysis for outcomes of FIM. There were significant heterogeneities among studies (*I*^2^ = 60.9%, *P* = .078), so we used a random-effects model. The pooled meta-analysis showed that technology-assisted rehabilitation were not significantly superior to usual care (SMD 0.53, 95% CI −0.01 to 1.08, *P* = .056). Subgroup analysis showed that telerehabilitation significantly improved the FIM score (SMD 1.26, 95% CI 0.48 to 2.03, *P* = .002) (Fig. 4).

#### 3.3.3. ADL.

**Figure 5.**
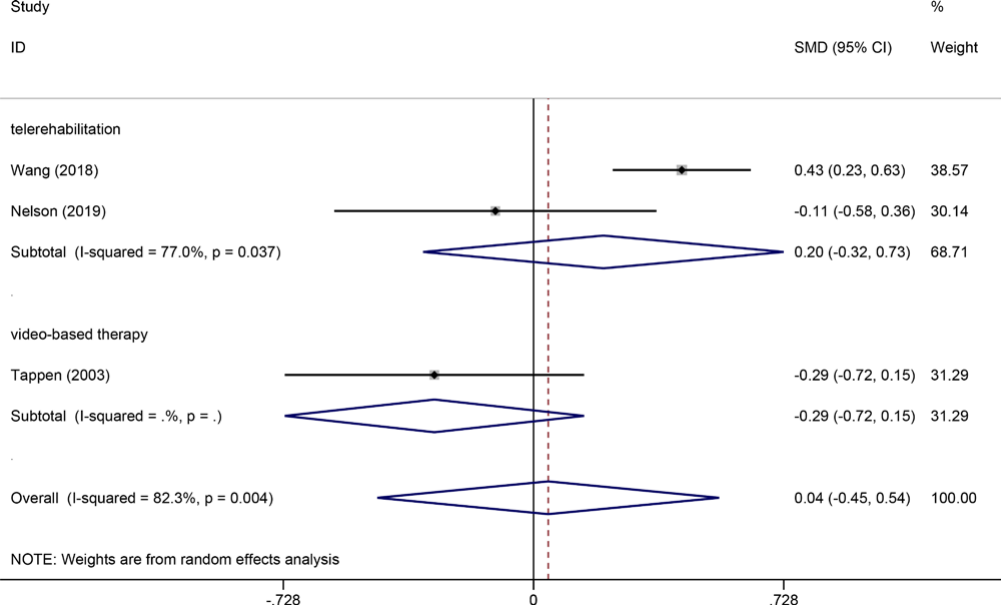
Forest plot of ADL. ADL = activities of daily living, CI = confidence interval, SMD = standardized mean difference.

Three studies reported on ADL outcomes. The random-effects meta-analysis revealed that compared with usual care, technology-assisted rehabilitation showed no significant difference in ADL (SMD 0.04, 95% CI −0.45 to 0.54, *P* = .886, *I*^2^ = 82.3%) (Fig. 5).

#### 3.3.4. Mental health.

**Figure 6.**
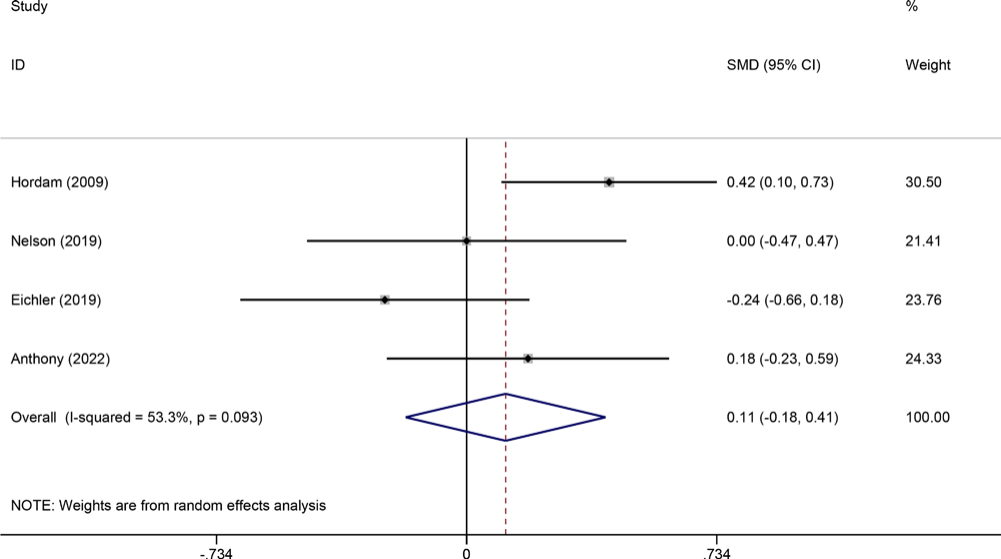
Forest plot of Mental Health. CI = confidence interval, SMD = standardized mean difference.

Four studies measured the effects of telerehabilitation on mental health. The pooled meta-analysis showed no significant effects on mental health between the telerehabilitation and control groups (SMD 0.11, 95% CI −0.18 to 0.41, *P* = .446, *I*^2^ = 53.3%) (Fig. 6).

#### 3.3.5. Physical health.

**Figure 7.**
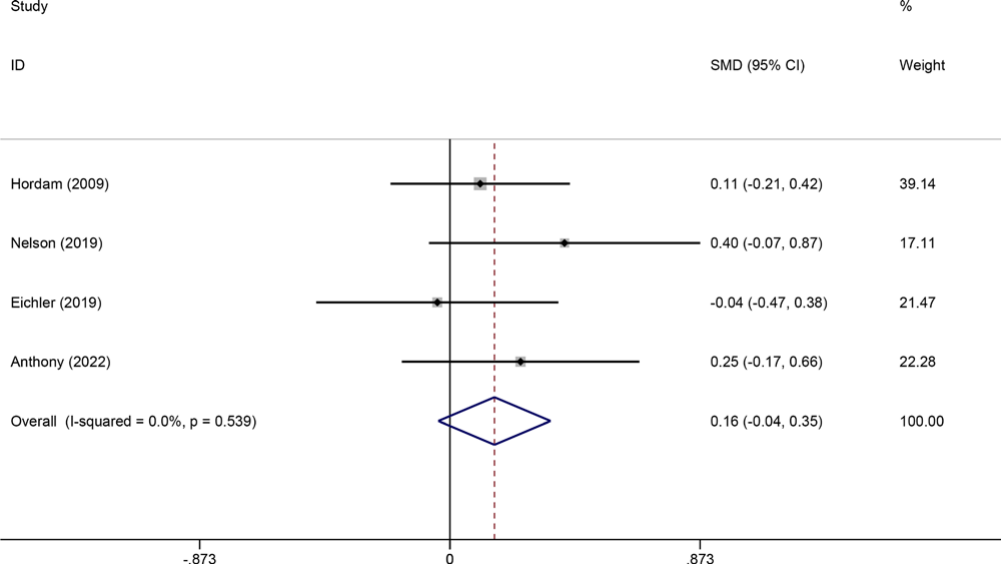
Forest plot of Physical Health. CI = confidence interval, SMD = standardized mean difference.

Four studies reported data on physical health. The pooled meta-analysis showed that telerehabilitation was not significantly superior to usual care (SMD −0.23, 95% CI −0.60 to 0.13, *P* = .204) (Fig. 7).

#### 3.3.6. TUG.

Two studies reported data on TUG. A fixed-effect model of analysis was used due to low heterogeneity among the studies (*I*^2^ = 0%). The meta-analysis showed no obvious difference in TUG performance telerehabilitation and control groups (SMD 0.16, 95% CI −0.04 to 0.35, *P* = .120, *I*^2^ = 0%).

#### 3.3.7. Walk test.

**Figure 8.**
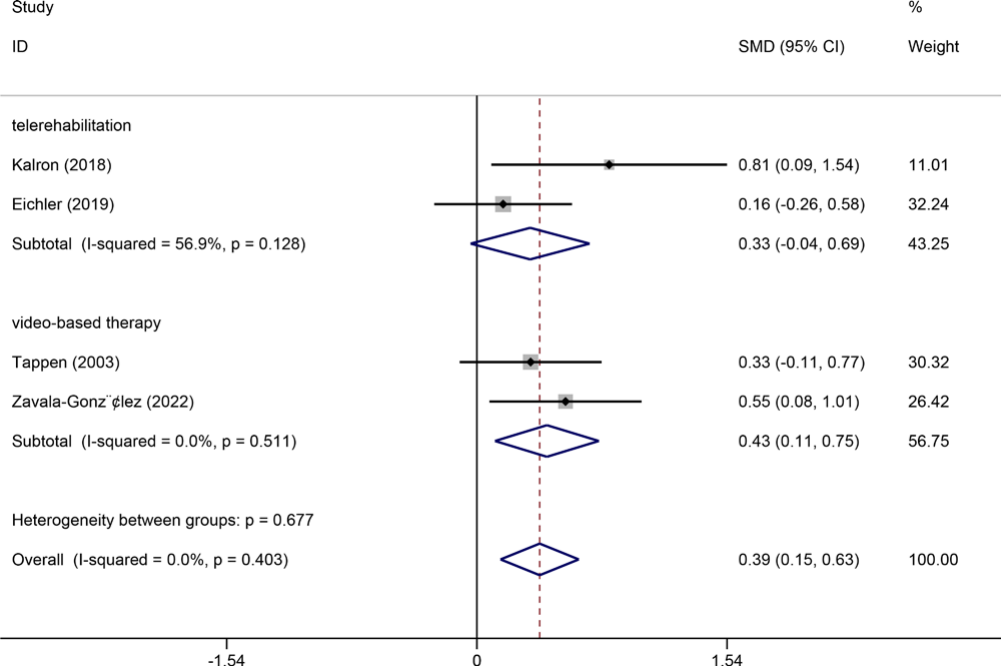
Forest plot of walk test. CI = confidence interval, SMD = standardized mean difference.

Four studies were included in the meta-analysis for outcomes of the walk test. A fixed-effect model of analysis was used due to low heterogeneity among the studies (*I*^2^ = 0%). The pooled meta-analysis showed that technology-assisted rehabilitation significantly improved the walk test results (SMD 0.39, 95% CI 0.15 to 0.63, *P* = .002). Subgroup analysis showed that telerehabilitation was not significantly superior to the usual car. In contrast, video-based therapy could significantly improve the walk test results (Fig. 8).

#### 3.3.8. HOOS JR.

The HOOS JR function was evaluated in 2 studies. A fixed-effect model of analysis was used due to low heterogeneity among the studies (*I*^2^ = 0%). The meta-analysis showed no obvious difference in HOOS JR between the 2 groups (SMD 0.18, 95% CI −0.16 to 0.52, *P* = .309).

## 4. Discussion

An early rehabilitation program is essential for functional recovery following THA.^[27]^ As the demand for rehabilitation of discharged THA patients has increased, the workload of medical personnel has been significantly increased.^[28]^ In recent years, the availability of low-cost internet and communication technology has encouraged the use of technology-assisted rehabilitation in clinical settings.^[29]^

While community-based medical care is currently being established, it is difficult for patients in rural or remote areas to obtain rehabilitation treatment through outpatient physiotherapy or family follow-up due to geographical isolation or inadequate community services. Alternative rehabilitation approaches are necessary. In previous studies, telerehabilitation has been found to be beneficial in a variety of postoperative orthopedic conditions; however, much of the research has examined the effects of telerehabilitation on patients with total knee replacements.^[30,31]^ The effectiveness of telerehabilitation techniques in THA patients has been compared with traditional face-to-face rehabilitation techniques in several randomized trials. Several studies reported positive results; however, the generalizability of the findings was limited by small samples and a variety of outcomes. The purpose of this study was to synthesize the current evidence regarding the effect of technology-assisted rehabilitation on patients with THA. Based on our findings, technology-assisted rehabilitation improved the Harris score and the walk test results more effectively than conventional rehabilitation care.

A new option for early rehabilitation is a systematic program of rehabilitation in a hospital and a home rehabilitation program supervised remotely.^[32]^ It is feasible for patients to study rehabilitation courses under the remote guidance of rehabilitation teachers from home, benefit from more intensive and autonomous rehabilitation, and participate in therapeutic exercises with greater ease and safety. In comparison to conventional rehabilitation therapy, telerehabilitation showed improvements in physical activity and functional status in TKA patients.^[33]^ The results of our meta-analysis indicated that telerehabilitation was associated with a greater Harris score and higher FIM in THA patients than conventional rehabilitation.

Virtual reality has gained popularity in rehabilitation over the years, showing promising results in reducing falls and improving balance and functionality.^[34]^ Several studies have reported that VR is beneficial to rehabilitation following TKR.^[35]^ Recent meta-analyses have shown that compared to traditional rehabilitation, VR-based rehabilitation improved functional outcomes 6 months postoperatively.^[36]^ There is, however, very limited evidence in support of the use of THA. The study by Zavala-González et al found that patients receiving the VR program had better performance on WOMAC, Berg, and 6-minute walk tests. In contrast, Fascio et al showed that compared to the traditional rehabilitation program, VR-based home rehabilitation resulted in similar improvements in functional outcomes. Our study showed that a video-based therapy programs after THA might be able to improve the walk test results compared to a traditional rehabilitation program. Although some interventions are provided at the patient home, the cost and availability of therapists remain a barrier to access. Since videoconferencing capabilities have become more widely available on mobile phones and laptops, it is anticipated that most interventions will be remote delivery soon. Therefore, VR-based rehabilitation can remove these barriers since patients can access and perform therapy at home without a therapist being present.

This meta-analysis has some limitations, which should be considered when interpreting the results. Firstly, the impact of publication bias could not assess owing to too few studies. Second, it may not be appropriate to generalize the results as the studies were conducted in different healthcare settings with patients of varying socioeconomic status. Third, the sample sizes were relatively small in the included studies, which might lead to bias in the pooling of effects. Fourth, due to a lack of long-term follow-up data, this study cannot determine the long-term effectiveness of technology-assisted rehabilitation. Fifth, there was heterogeneity among some outcome measures in this study. This may be related to differences in technology-assisted rehabilitation, including intervention duration, intensity, frequency, content, and so on. Lastly, since the outcome measures varied and statistical heterogeneity exists among the studies, it is not possible to make a strict recommendation for methods that promote functional improvements.

## 5. Conclusion

The results of this meta-analysis revealed that technology-assisted rehabilitation, especially telerehabilitation, improved the physical function of patients following THR when compared to conventional rehabilitation. The study is limited by the availability of high-quality evidence, however, more robust studies are needed to validate the long-term efficacy and safety of innovative technology-assisted training strategies.

## Author contributions

**Conceptualization:** Qiuhua Sun.

**Data curation:** Lingyun Wu.

**Formal analysis:** Lingyun Wu, Xiaoyan Li, Lijiangshan Hua.

**Investigation:** Lingyun Wu, Xiaoyan Li.

**Software:** Lingyun Wu, Xiaoyan Li.

**Supervision:** Qiuhua Sun.

**Validation:** Qiuhua Sun.

**Writing – original draft:** Lingyun Wu, Xiaoyan Li, Lijiangshan Hua.

**Writing – review & editing:** Lingyun Wu, Xiaoyan Li, Lijiangshan Hua.

## Supplementary Material


