# Supplementary material for: Effects of technology-assisted rehabilitation for patients with hip arthroplasty: A meta-analysis
Source: Medicine (Baltimore). 2023 Nov 10;102(45):e35921. doi: 10.1097/MD.0000000000035921 (PMC10637488; doi:10.1097/MD.0000000000035921)
Supplement: Supplementary file 1 [file medi-102-e35921-s001.docx]

**Supplementary Table1. Search Strategy**

| **PubMed** | |
| --- | --- |
| #1 | "arthroplasty, replacement, hip"[MeSH Terms] OR ("arthroplasty"[All Fields] AND "replacement"[All Fields] AND "hip"[All Fields]) OR "hip replacement arthroplasty"[All Fields] OR ("hip"[All Fields] AND "replacement"[All Fields]) OR "hip replacement"[All Fields] OR (("hip"[MeSH Terms] OR "hip"[All Fields]) AND ("arthroplasty"[MeSH Terms] OR "arthroplasty"[All Fields] OR "arthroplasties"[All Fields])) |
| #2 | "random allocation"[MeSH Terms] OR ("random"[All Fields] AND "allocation"[All Fields]) OR "random allocation"[All Fields] OR "randomization"[All Fields] OR "randomized"[All Fields] OR "random"[All Fields] OR "randomisation"[All Fields] OR "randomisations"[All Fields] OR "randomise"[All Fields] OR "randomised"[All Fields] OR "randomising"[All Fields] OR "randomizations"[All Fields] OR "randomize"[All Fields] OR "randomizes"[All Fields] OR "randomizing"[All Fields] OR "randomness"[All Fields] OR "randoms"[All Fields] OR ("random allocation"[MeSH Terms] OR ("random"[All Fields] AND "allocation"[All Fields]) OR "random allocation"[All Fields]) OR ("randomized controlled trial"[Publication Type] OR "randomized controlled trials as topic"[MeSH Terms] OR "randomized controlled trials"[All Fields] OR "randomised controlled trials"[All Fields]) |
| #3 | "telecare"[All Fields] OR ("telerehabilitation"[MeSH Terms] OR "telerehabilitation"[All Fields] OR ("remote"[All Fields] AND "rehabilitation"[All Fields]) OR "remote rehabilitation"[All Fields]) OR ("telerehabilitation"[MeSH Terms] OR "telerehabilitation"[All Fields] OR ("virtual"[All Fields] AND "rehabilitation"[All Fields]) OR "virtual rehabilitation"[All Fields]) OR ("telerehabilitation"[MeSH Terms] OR "telerehabilitation"[All Fields]) OR ("virtual reality"[MeSH Terms] OR ("virtual"[All Fields] AND "reality"[All Fields]) OR "virtual reality"[All Fields]) OR ("exergamers"[All Fields] OR "exergaming"[MeSH Terms] OR "exergaming"[All Fields] OR "exergame"[All Fields] OR "exergames"[All Fields]) OR ("game s"[All Fields] OR "games"[All Fields] OR "gaming"[All Fields]) OR ("video s"[All Fields] OR "videoed"[All Fields] OR "videotape recording"[MeSH Terms] OR ("videotape"[All Fields] AND "recording"[All Fields]) OR "videotape recording"[All Fields] OR "video"[All Fields] OR "videos"[All Fields]) OR ("telemedicine"[MeSH Terms] OR "telemedicine"[All Fields] OR "ehealth"[All Fields]) OR ("telemedicine"[MeSH Terms] OR "telemedicine"[All Fields] OR ("mobile"[All Fields] AND "health"[All Fields]) OR "mobile health"[All Fields]) OR ("internet based intervention"[MeSH Terms] OR ("internet based"[All Fields] AND "intervention"[All Fields]) OR "internet based intervention"[All Fields] OR ("internet"[All Fields] AND "based"[All Fields] AND "intervention"[All Fields]) OR "internet based intervention"[All Fields]) |
| #4 | #1 AND #2 AND #3 |
| **Embase** | |
| #1 | 'hip replacement'/exp OR 'hip replacement' OR (('hip'/exp OR hip) AND ('replacement'/exp OR replacement)) OR 'hip arthroplasty'/exp OR 'hip arthroplasty' OR (('hip'/exp OR hip) AND ('arthroplasty'/exp OR arthroplasty)) |
| #2 | random OR 'random allocation'/exp OR 'random allocation' OR (random AND allocation) OR 'randomized controlled trials'/exp OR 'randomized controlled trials' OR (randomized AND controlled AND trials) |
| #3 | 'telecare'/exp OR telecare OR 'remote rehabilitation'/exp OR 'remote rehabilitation' OR (remote AND ('rehabilitation'/exp OR rehabilitation)) OR 'virtual rehabilitation'/exp OR 'virtual rehabilitation' OR (virtual AND ('rehabilitation'/exp OR rehabilitation)) OR 'telerehabilitation'/exp OR telerehabilitation OR 'virtual reality'/exp OR 'virtual reality' OR (virtual AND ('reality'/exp OR reality)) OR exergames OR games OR 'video'/exp OR video OR 'ehealth'/exp OR ehealth OR 'mobile health'/exp OR 'mobile health' OR (mobile AND ('health'/exp OR health)) OR 'internet-based intervention'/exp OR 'internet-based intervention' OR ('internet based' AND ('intervention'/exp OR intervention)) |
| #4 | #1 AND #2 AND #3 |
| **Cochrane** | |
| #1 | MeSH descriptor: [Arthroplasty, Replacement, Hip] explode all trees OR hip replacement:ti,ab,kw OR hip arthroplasty:ti,ab,kw |
| #2 | MeSH descriptor: [randomized controlled trials] explode all trees OR MeSH descriptor: [random allocation] explode all trees OR random:ti,ab,kw OR random allocation:ti,ab,kw OR randomized controlled trials:ti,ab,kw |
| #3 | MeSH descriptor: [eHealth] explode all trees OR MeSH descriptor: [telerehabilitation] explode all trees OR MeSH descriptor: [virtual rehabilitation] explode all trees OR MeSH descriptor: [Telemedicine] explode all trees OR MeSH descriptor: [virtual reality] explode all trees OR MeSH descriptor: [Telemedicine] explode all trees OR MeSH descriptor: [Video Games] explode all trees OR MeSH descriptor: [internet-based intervention] explode all trees OR MeSH descriptor: [Video] explode all trees OR telecare:ti,ab,kw OR remote rehabilitation:ti,ab,kw OR virtual rehabilitation OR telerehabilitation:ti,ab,kw OR virtual reality:ti,ab,kw OR exergames:ti,ab,kw OR games:ti,ab,kw OR video:ti,ab,kw OR eHealth:ti,ab,kw OR mobile health:ti,ab,kw OR internet-based intervention:ti,ab,kw |
| #4 | #1 AND #2 AND #3 |
| Web of science | |
| #1 | (ALL=(hip replacement)) OR ALL=(hip arthroplasty) |
| #2 | ((ALL=(random)) OR ALL=(randomized controlled trials)) OR ALL=(random allocation) |
| #3 | ((((((((((ALL=(telecare )) OR ALL=(remote rehabilitation)) OR ALL=(virtual rehabilitation)) OR ALL=(telerehabilitation)) OR ALL=(virtual reality)) OR ALL=(exergames)) OR ALL=(games)) OR ALL=(video)) OR ALL=(eHealth)) OR ALL=(mobile health)) OR ALL=(internet-based intervention) |
| #4 | #1 AND #2 AND #3 |
